# Effect of Adrenergic Agonists on High-Fat Diet-Induced Hepatic Steatosis in Mice

**DOI:** 10.3390/ijms21249392

**Published:** 2020-12-10

**Authors:** Yukiomi Nakade, Rena Kitano, Taeko Yamauchi, Satoshi Kimoto, Kazumasa Sakamoto, Tadahisa Inoue, Yuji Kobayashi, Tomohiko Ohashi, Yoshio Sumida, Kiyoaki Ito, Masashi Yoneda

**Affiliations:** Division of Gastroenterology and Hepatology, Department of Internal Medicine, Aichi Medical University, Nagakute 480-1195, Aichi, Japan; kitano.rena.035@mail.aichi-med-u.ac.jp (R.K.); tyamauch@aichi-med-u.ac.jp (T.Y.); kimoto.satoshi.146@mail.aichi-med-u.ac.jp (S.K.); sakamoto.kazumasa.041@mail.aichi-med-u.ac.jp (K.S.); tinoue-tag@umin.ac.jp (T.I.); kobayashi.yuuji.572@mail.aichi-med-u.ac.jp (Y.K.); oohashi.tomohiko.415@mail.aichi-med-u.ac.jp (T.O.); sumida.yoshio.500@mail.aichi-med-u.ac.jp (Y.S.); kito@aichi-med-u.ac.jp (K.I.); yoneda@aichi-med-u.ac.jp (M.Y.)

**Keywords:** phenylephrine, isoproterenol, steatosis, β-oxidation, autophagy

## Abstract

The autonomic nervous system, consisting of sympathetic and parasympathetic branches, plays an important role in regulating metabolic homeostasis. The sympathetic nervous system (SNS) regulates hepatic lipid metabolism by regulating adrenergic receptor activation, resulting in the stimulation of hepatic very-low-density lipoprotein-triglyceride (TG) production in vivo. However, only a few studies on the relationship between SNS and hepatic steatosis have been reported. Here, we investigate the effect of adrenergic receptor agonists on hepatic steatosis in mice fed a high-fat diet (HFD). The α-adrenergic receptor agonist phenylephrine (10 mg/kg/d) or the β-adrenergic receptor agonist isoproterenol (30 mg/kg/d) was coadministered with HFD to male mice. After five weeks, hepatic steatosis, TG levels, and hepatic fat metabolism-related biomarkers were examined. HFD treatment induced hepatic steatosis, and cotreatment with phenylephrine, but not isoproterenol, attenuated this effect. Phenylephrine administration upregulated the mRNA levels of hepatic peroxisome proliferator-activated receptor alpha and its target genes (such as carnitine palmitoyltransferase 1) and increased hepatic β-hydroxybutyrate levels. Additionally, phenylephrine treatment increased the expression of the autophagosomal marker LC3-II but decreased that of p62, which is selectively degraded during autophagy. These results indicate that phenylephrine inhibits hepatic steatosis through stimulation of β-oxidation and autophagy in the liver.

## 1. Introduction

Nonalcoholic fatty liver disease (NAFLD) is considered the hepatic manifestation of metabolic syndrome and metabolic comorbidities associated with obesity, type 2 diabetes, hyperlipidemia, and hypertension [1]. NAFLD is the leading cause of liver disease worldwide, prevalent in 25% of the global population [1]. A study of the histological course of NAFLD showed that approximately one-third of NAFLD patients display progression to liver fibrosis within three years, and 29% display fibrosis regression [2]. Thus, NAFLD does not always progress to liver fibrosis [2]. Although various adipose tissue-derived factors, gut-derived signals, and genetic factors are thought to contribute to the development of NAFLD, the pathogenesis of NAFLD progression remains to be elucidated [3].

The autonomic nervous system, which consists of sympathetic and parasympathetic branches, plays an important role in regulating metabolic homeostasis [4]. The sympathetic nervous system (SNS) regulates hepatic steatosis, which is involved in triglyceride (TG) metabolism by regulating the activation of adrenergic receptors. This activation results in the stimulation of hepatic very-low-density lipoprotein (VLDL)–TG production to increase plasma TG levels in vivo [5]. Furthermore, SNS stimulates VLDL–TG production in obese rats, and hepatic SNS denervation reduces VLDL–TG secretion [6]. Regarding the association between SNS and NAFLD, catecholamine is upregulated in human NAFLD and regulates the fibrotic function of hepatic stellate cells; however, only a few findings have been reported [7].

Previous reports have indicated that hepatic SNS activates the hepatic stellate cells involved in hepatic fibrosis [2]. Both α- and β-adrenergic receptor antagonists have been reported to affect liver injury models [8,9,10]. Regarding hepatic inflammation, the α-adrenergic receptor antagonist prazosin reduces liver injury, whereas the β-adrenergic receptor antagonist propranolol deteriorates liver injury [8,9,10]. Moreover, SNS is thought to regulate NAFLD. However, it remains to be elucidated whether adrenergic receptor agonists affect hepatic steatosis in NAFLD.

In the present study, we examine how α- and β-adrenergic receptor agonists affect hepatic steatosis in mice fed a high-fat diet (HFD). We demonstrate that α-adrenergic receptor agonists, but not β-adrenergic receptor agonists, reduce steatosis in mice by stimulating fatty acid oxidation and autophagy in the liver.

## 2. Results

### 2.1. Effect of Phenylephrine and Isoproterenol on Hepatic Steatosis and VLDL–TG Secretion

Body weight increased significantly in HFD-fed mice compared with that of control mice (Table 1). HFD increased body weight, which did not change after cotreatment with phenylephrine or isoproterenol (Table 1). HFD-fed mice had a higher subepididymal fat weight than control mice; this weight was further increased by phenylephrine treatment but decreased by isoproterenol treatment (Table 1).

**Figure 1:**
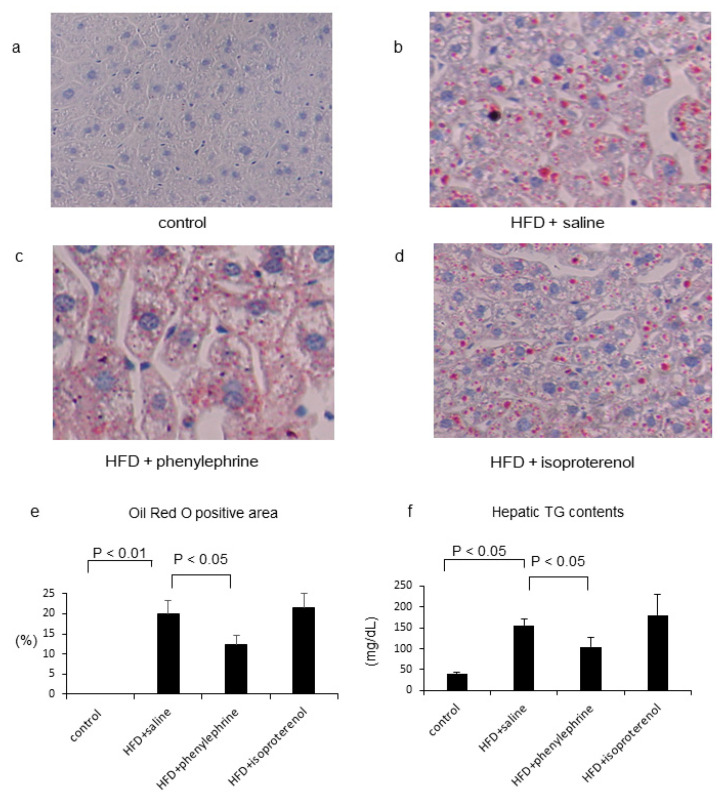
Representative images of liver tissues stained with Oil Red O. Mice were fed with control diet (*n* = 5) (**a**), high-fat diet (HFD; *n* = 6) (**b**), HFD with phenylephrine (*n* = 6) (**c**), or HFD with isoproterenol (*n* = 7) (**d**). Original magnification, 100×. The Oil Red O-positive area was quantified in five randomly selected fields per section (**e**). The percentage of Oil Red O-positive area was measured with a computerized image analysis system using Image-Pro Plus version 4.5. Hepatic triglyceride (TG) contents (mg/dL) in the respective groups (**f**). Statistical analysis was performed using analysis of variance (ANOVA). Data are expressed as means ± standard error (SE).

Oil Red O staining results showed that HFD-fed mice had an increase in the Oil Red O-positive area, which was decreased by phenylephrine treatment but remained unchanged after isoproterenol treatment (Figure 1a–e). Moreover, HFD-fed mice showed markedly increased hepatic TG levels compared with that of control mice, which was decreased by phenylephrine treatment but remained unchanged after isoproterenol treatment (Figure 1a–e). HFD-fed mice also showed significantly increased hepatic free fatty acid (FFA) levels, which were significantly decreased by phenylephrine treatment but remained unchanged after isoproterenol treatment (Table 2). On the other hand, HFD treatment did not alter serum TG levels, which were increased by phenylephrine and decreased by isoproterenol cotreatment (Table 2). In addition, HFD treatment did not alter the serum FFA level, which was increased by cotreatment with phenylephrine but remained unchanged by isoproterenol cotreatment (Table 2). Compared to control-diet-fed mice, HFD-fed mice displayed increased fasting blood glucose (FBG) levels, which remained unchanged after treatment with phenylephrine or isoproterenol (Table 1). Furthermore, HFD-mice displayed no changes in serum immunoreactive insulin (IRI) levels, which remained unaltered by phenylephrine treatment but were significantly reduced by isoproterenol treatment. HFD treatment significantly increased serum cholesterol levels, which remained unchanged by phenylephrine or isoproterenol cotreatment (Table 2).

### 2.2. Effect of Phenylephrine or Isoproterenol on Hepatic Fat Metabolism-Related Genes

**Figure 2:**
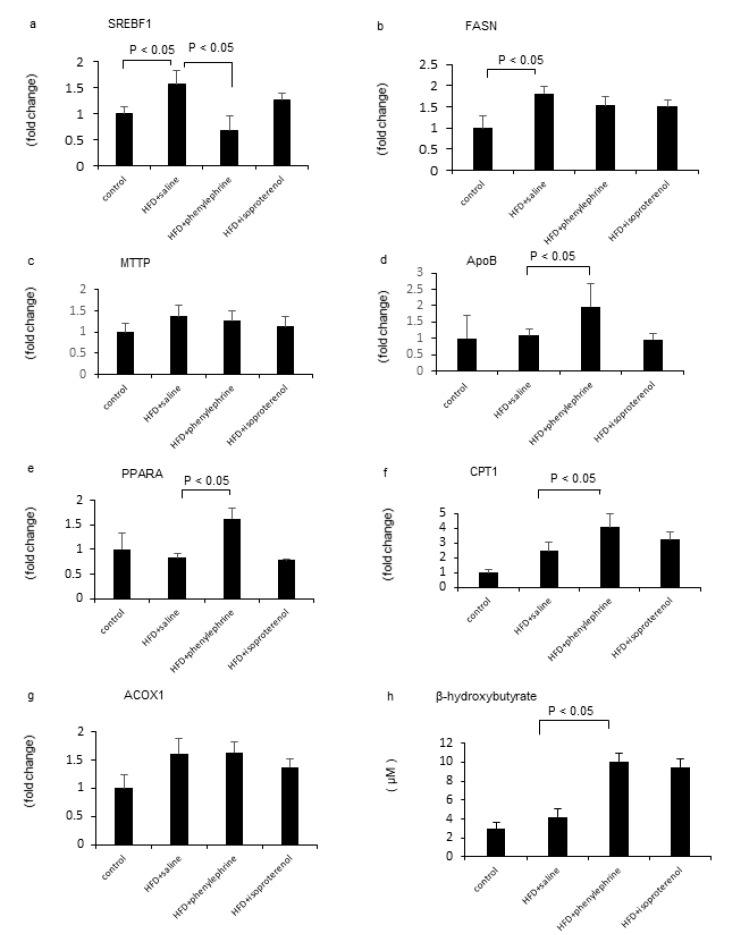
Evaluation of hepatic lipid metabolism-related genes. Relative mRNA expression levels of SREBF1 (**a**), FASN (**b**), MTTP (**c**), ApoB (**d**), PPARα (**e**), CPT1 (**f**), and ACOX1 (**g**) were evaluated. Hepatic β-hydroxybutyrate contents were evaluated using ELISA (**h**). Statistical analysis was performed using ANOVA. Data are expressed as means ± SE.

HFD treatment significantly increased the mRNA levels of hepatic sterol regulatory element-binding transcription factor 1 (SREBF1) and fatty acid synthase (FASN; Figure 2a,b) but did not change those of hepatic microsomal triglyceride transfer protein (MTTP), apolipoprotein B (ApoB), peroxisome proliferator-activated receptor alpha (PPARα), carnitine palmitoyltransferase 1 (CPT1), and acyl-coenzyme A oxidase 1 (ACOX1; Figure 2c–g). Phenylephrine attenuated the HDF-induced increase in the mRNA level of SREBF1 (Figure 2a) but did not change that of hepatic FASN (Figure 2b). In addition, phenylephrine treatment did not change the mRNA levels of hepatic MTTP and ACOX1 in HFD-fed mice, but it increased those of hepatic ApoB, PPARα, and CPT1 (Figure 2c–g). On the other hand, isoproterenol treatment did not alter the mRNA levels of hepatic SREBF1, FASN, MTTP, ApoB, PPARα, CPT1, and ACOX1 in HFD-fed mice. Hepatic β-hydroxybutyrate levels in HFD-fed mice were significantly increased by phenylephrine treatment but were not significantly increased by isoproterenol treatment (Figure 2h).

### 2.3. Effect of Phenylephrine and Isoproterenol on VLDL–TG Secretion

**Figure 3:**
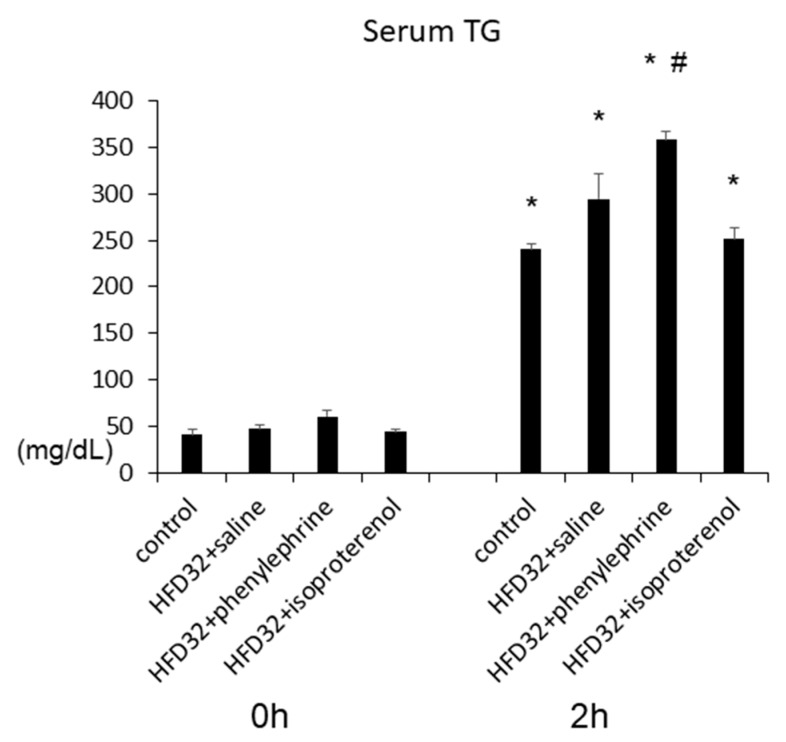
Evaluation of hepatic VLDL–TG secretion by using TR-1339. Five weeks after the commencement of control diet (*n* = 4), HFD (*n* = 4), HFD with phenylephrine (*n* = 4), and HFD with isoproterenol (*n* = 4), these four cohorts of mice were fasted overnight and then injected with 0.2 mL of Triton WR-1339 (10% (*v*/*v*) solution). Serum TG levels were measured at 0 and 2 h. Statistical analysis was performed using ANOVA. Data are expressed as means ± SE. * *p* < 0.01 for each group compared to those at 0 h. ^#^ *p* < 0.05 for HFD + phenylephrine group compared to HFD + saline group at 2 h.

Hepatic VLDL secretion was evaluated by measuring serum TG levels after injections of Triton WR-1339, which inhibits lipoprotein lipase activity in fasting mice [9]. Results showed that at 2 h after Triton WR-1339 injections, serum TG levels were significantly increased in both control-diet-fed and HFD-fed mice. Moreover, treatment with phenylephrine, but not isoproterenol, increased serum TG levels significantly in HFD-fed mice (Figure 3).

### 2.4. Hepatic Inflammatory Responses by Phenylephrine and Isoproterenol

**Figure 4:**
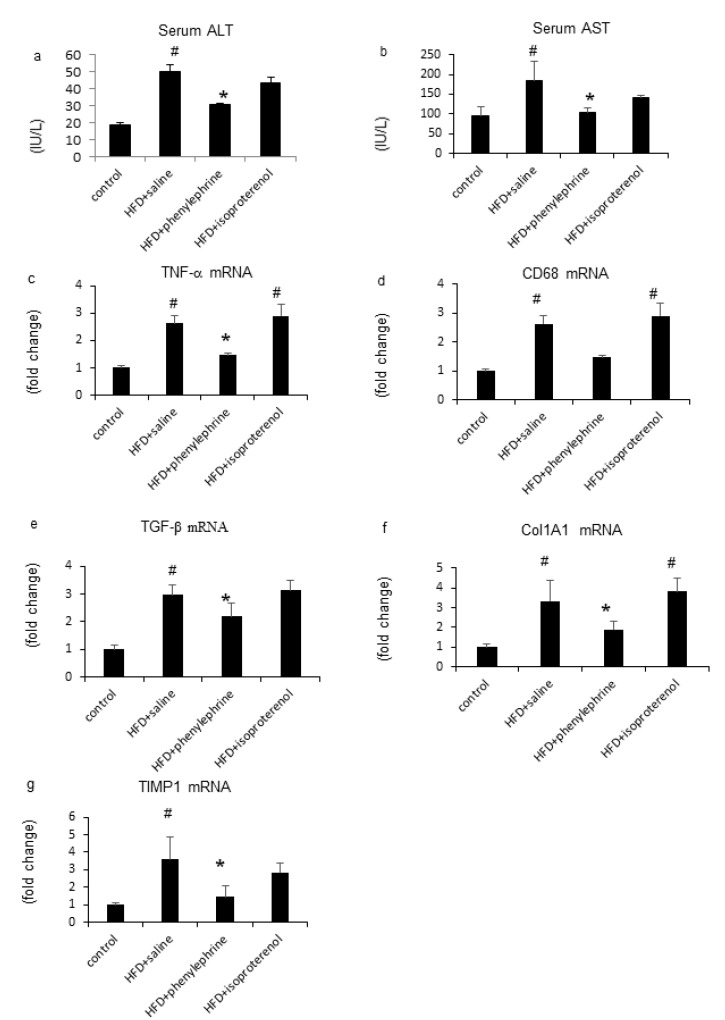
Evaluation of hepatic inflammation-related parameters. Serum alanine aminotransferase (ALT) levels (**a**) and aspartate aminotransferase (AST) levels (**b**). Relative mRNA expression levels of TNF-α (**c**), CD68 (**d**), TGF-β (**e**), Col1A1 (**f**), and TIMP1 (**g**) were evaluated in the liver. Statistical analysis was performed using ANOVA. Data are expressed as means ± SE. ^#^ *p* < 0.05 for HFD with saline or isoproterenol group compared with the control group. * *p* < 0.05 for HFD with phenylephrine group compared to HFD with the saline group.

HFD treatment increased the mRNA levels of serum alanine aminotransferase (ALT), aspartate aminotransferase (AST), and hepatic tumor necrosis factor-α (TNF-α), which remained unchanged following isoproterenol cotreatment but decreased after phenylephrine cotreatment (Figure 4a–c). In addition, HFD treatment increased the mRNA level of cluster of differentiation 68 (CD68), which decreased following phenylephrine cotreatment but not after isoproterenol cotreatment (Figure 4d). HFD treatment also increased the mRNA level of hepatic transforming growth factor (TGF)-β, which decreased following phenylephrine cotreatment but not after isoproterenol cotreatment (Figure 4e). Moreover, HFD-fed mice displayed an increase in the mRNA levels of hepatic collagen 1 (Col1A1) and tissue inhibitor of metalloproteinase 1 (TIMP-1), which were attenuated following phenylephrine treatment but not after isoproterenol treatment (Figure 4f,g).

### 2.5. Enhancement of Autophagy by Phenylephrine and Isoproterenol Via Regulation of Autophagy-Related 7 (Atg7) and Sequestosome-1 (SQSTM1)

**Figure 5:**
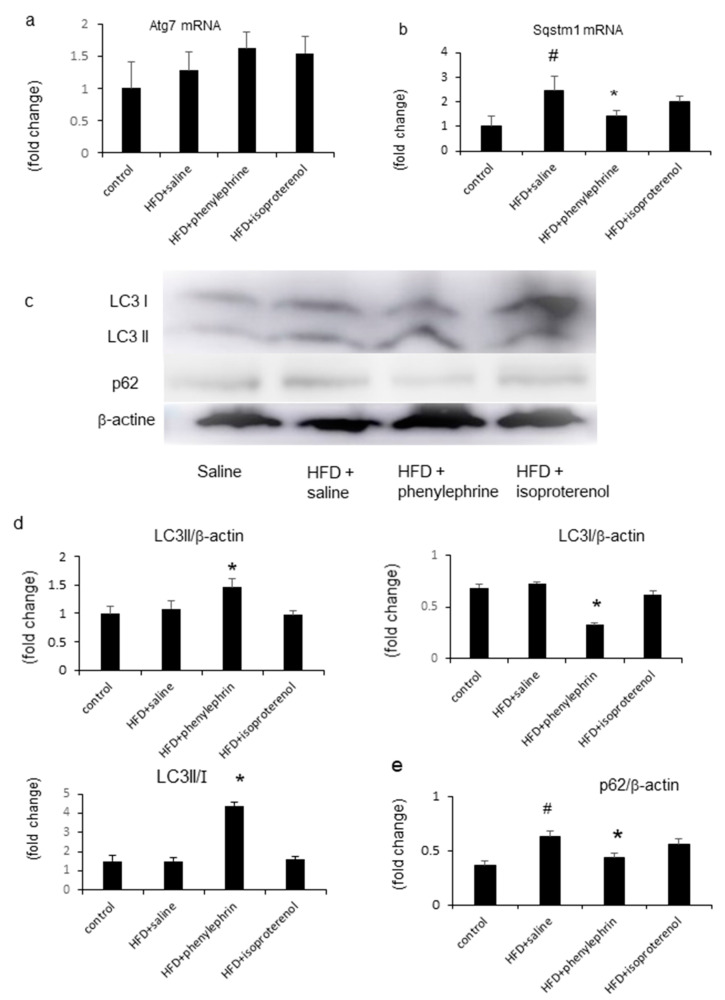
Evaluation of hepatic autophagy-related parameters. Relative mRNA expressions of Atg7 (**a**) and SQSTM1 (**b**). Western blotting autoradiograph images (**c**) and levels (**d**,**e**) of LC3 and p62 autophagy-related proteins in mice. Statistical analysis was performed using ANOVA. Data are expressed as means ± SE. ^#^ *p* < 0.05 for HFD with saline group compared with the control group. * *p* < 0.05 for HFD with phenylephrine group compared to HFD with the saline group.

Activation of autophagy contributes to decreasing fat storage in cells and tissues. Here, HFD treatment did not affect the mRNA level of hepatic Atg7, which tended to increase after cotreatment with phenylephrine or isoproterenol (Figure 5a). However, HFD treatment significantly increased the mRNA level of hepatic SQSTM1, which was significantly decreased by phenylephrine treatment but remained unaffected by isoproterenol treatment (Figure 5b). Western blot analysis showed that HFD treatment had no effect on microtubule-associated protein light chain 3 (LC3)-II protein expression (Figure 5c,d); however, cotreatment with phenylephrine, but not isoproterenol, increased LC3-II protein expression (Figure 5d). HFD treatment also did not alter the protein expression of LC3-I, which was decreased by cotreatment with phenylephrine, but not isoproterenol (Figure 5c,d). The LC3-II/-I ratio in HFD-fed mice was significantly increased by phenylephrine but not isoproterenol treatment (Figure 5c,d). Moreover, HFD treatment significantly increased p62 protein expression, which was significantly attenuated by cotreatment with phenylephrine but not isoproterenol (Figure 5e).

### 2.6. Hepatic Adrenergic Receptors mRNA and Proteins Mediated by Phenylephrine and Isoproterenol

**Figure 6:**
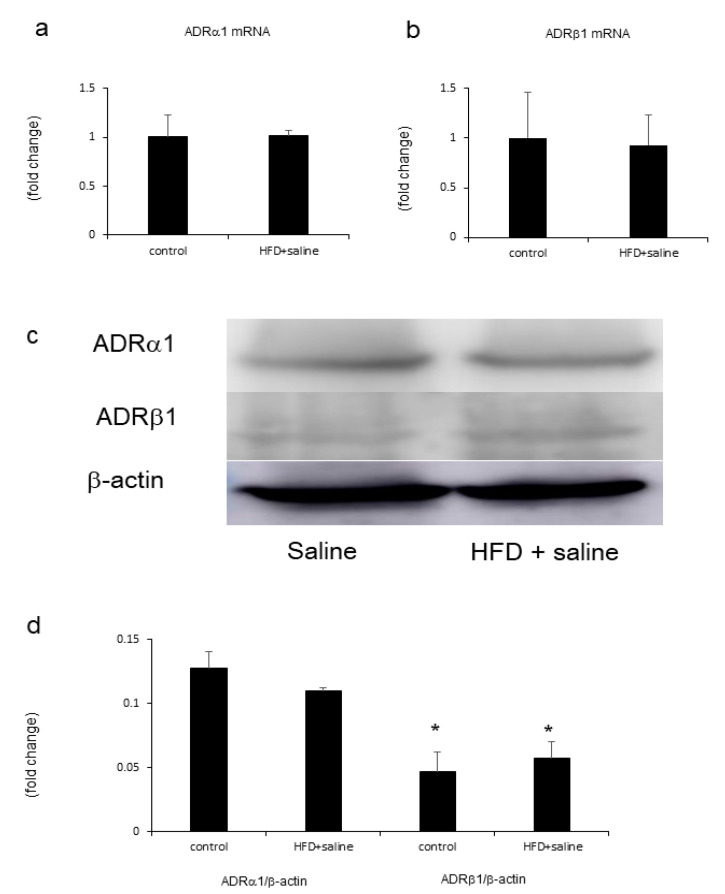
Evaluation of the mRNA expression of hepatic adrenergic receptors. (**b**). Relative mRNA expression levels of ADRα1 (**a**) and ADRβ1 (**b**) were evaluated in the liver. Western blotting images (**c**) and levels (**d**) of ADRα1 and ADRβ1 in the liver. Statistical analysis was performed using Student’s *t*-test or analysis of variance (ANOVA) from different treatment groups. Data are expressed as means ± SE. * *p* < 0.05 for ADRα1/β-actin compared to ADRβ1/β-actin in the respective control and HFD with the saline group.

Both control and HFD treatment produced the mRNA and protein of hepatic adrenergic receptors α1 (ADRα1) and β1 (ADRβ1) (Figure 6). These proteins’ levels were not significantly changed by HFD. However, ADRα1 protein levels were higher than ADRβ1 protein levels in the liver (Figure 6).

## 3. Discussion

In the current study, we investigated how α- and β-adrenergic receptor agonists affect HFD-induced hepatic steatosis using an NAFLD mouse model. We demonstrated that administration of phenylephrine reduced HFD-induced hepatic steatosis, as indicated by hepatic TG contents. However, administration of isoproterenol did not affect hepatic steatosis. Hence, only phenylephrine stimulated hepatic fatty acid oxidation and autophagy.

Previously, with regard to hepatic fat metabolism, FFA influx, hepatic de novo lipogenesis, β-oxidation, and VLDL-TG production have been shown to be impaired in NAFLD and NASH models [11,12,13]. Hepatic influx of FFA and impairment of VLDL secretion play important roles in NASH progression [11]. In humans with NASH, hepatic lipids are predominantly taken up by inappropriate lipolysis [14]. Here, we showed that phenylephrine treatment significantly decreased hepatic TG contents and increased serum TG levels in HFD-fed mice. However, treatment with isoproterenol had no effect on hepatic and serum TG levels. Furthermore, after inhibition of serum lipoprotein degradation, phenylephrine, but not isoproterenol, increased serum TG levels. These results indicate that phenylephrine reduces hepatic TG levels, resulting in an increase of VLDL–TG levels.

Next, we analyzed hepatic gene expression with regard to lipid metabolism in the liver. ApoB and MTTP are proteins related to the production of VLDL and the excretion of TG from hepatocytes [15]. Here, MTTP mRNA levels were not altered in HFD-fed mice and remained unchanged following phenylephrine and isoproterenol treatment. ApoB mRNA levels also remained unchanged in HFD-fed mice but increased significantly following phenylephrine treatment. The HFD-induced increase in the mRNA level of hepatic SREBF1 was due to hepatic de novo lipogenesis; this effect was significantly attenuated by treatment with phenylephrine, but not isoproterenol. Hepatic β-oxidation is an energy-consuming process and involves various genes such as ACOX1 and CPT1 [16,17]. PPARα is expressed in the liver and is involved in hepatic β-oxidation [18]. PPARα gene expression is associated with histological treatment response in NASH [19]. Administration of PPARα agonists attenuates HFD-induced hepatic TG accumulation [20] and upregulates the expression of β-oxidation enzymes, thereby reducing hepatic TG levels in mice [21]. We showed that the mRNA levels of hepatic ACOX1, CPT1, and PPARα were not significantly altered in HFD-fed mice but were significantly increased by phenylephrine treatment. Furthermore, the hepatic β-hydroxybutyrate level was increased in HFD-fed mice following phenylephrine treatment. These results indicate that phenylephrine inhibits the HFD-induced increase in TG levels by stimulating β-oxidation in the liver. Although the hepatic FFA level decreased after phenylephrine treatment, serum TG and FFA levels increased. We showed that subepididymal fat weight tended to increase after phenylephrine treatment. Furthermore, phenylephrine significantly increased VLDL–TG excretion from the liver. This might explain the increase in the ApoB mRNA level, which contributes to VLDL–TG excretion from the liver. Thus, we speculate that subepididymal fat influences serum FFA and that hepatic TG excretion affects serum TG increase in mice.

Regarding the role of ADRα1 and ADRβ1 in the liver, fat accumulation was reported to be augmented by ADRβ overexpression in isolated hepatocytes [22]. On the other hand, the mRNA level of ADRβ1 decreased in subcutaneous fat cells in an age-dependent fashion [23], whereas that of ADRα1 increased [24]. However, the levels of ADRα1 and ADRβ1 in the mouse liver have not been investigated yet. We examined the involvement of hepatic ADRα1 and ADRβ1 in HFD-induced hepatic steatosis and found that the mRNA and protein of both receptors emerged in the liver. ADRα1 protein levels were higher than ADRβ1 protein levels in the liver. These results indicate that ADRα1 might be involved in the mechanism where phenylephrine affects liver steatosis.

Previous reports confirming β-adrenergic-responsive liver steatosis by experiments have shown that isoproterenol treatment of mice affects hepatic lipid content [22]. However, α-adrenergic-responsive liver steatosis remains to be elucidated. It is of interest to examine whether phenylephrine attenuates HFD-induced steatosis under continuous administration of α-adrenergic receptor antagonists.

In this study, hepatic inflammation- and fibrosis-related genes were upregulated by HFD. Assessment of the expression of hepatic inflammation-related genes in the liver revealed that the mRNA levels of hepatic TNF-α, CD68, TGF-β, and TIMP-1 significantly increased in HFD-fed mice. Further treatment with phenylephrine, but not isoproterenol, attenuated this HFD-induced increase. Previous reports have indicated that reduced TGF-β signaling attenuates steatohepatitis [25]. The altered abundance and composition of fat in the liver can modulate the biological activity of Kupffer cells, which involves the recruitment of macrophages and the augmentation of hepatic inflammation in vivo [26]. The amount of Kupffer cells are significantly increased in the liver during HFD [26]. This increase in Kupffer cells leads to the release of reactive oxygen species that enhance the sensitivity of Kupffer cells to hepatotoxin, which, in turn, stimulates the secretion of proinflammatory cytokines and chemokines such as TNF-α. HFD may increase the amount of Kupffer cells and levels of proinflammatory cytokines. HFD induces a phenotype in mice that is similar to the human disease, which is characterized by obesity [27]. Thus, phenylephrine might reduce the amounts of Kupffer cells and proinflammatory cytokines.

We also examined whether phenylephrine-induced inhibition of hepatic steatosis was associated with autophagy. Autophagy is initiated by the formation of a small membrane particle, called the autophagosome, which is generated through various cell signaling cascades involving autophagy-related genes and LC3 [28]. The interrelationship between β-oxidation and autophagy has been reported [29]. Knockdown of the autophagy-related gene Atg5 in hepatocytes was shown to increase TG levels and inhibit β-oxidation [29], indicating that inhibition of autophagy decreases β-oxidation in the liver. In the present study, hepatic Atg7 mRNA and LC3 protein levels were increased in HFD-fed mice, and treatment with phenylephrine or isoproterenol further increased these levels. In addition, HFD treatment increased the levels of hepatic SQSTM1 and p62, which are selectively degraded by autophagy; further treatment with phenylephrine, but not isoproterenol, attenuated this effect. These results indicate that hepatic autophagy is impaired in HFD-fed mice but can be improved by phenylephrine administration.

In conclusion, our data suggest that phenylephrine attenuates HFD-induced steatosis through the increase of VLDL–TG secretion and stimulation of β-oxidation and autophagy in the liver. This indicates that α-adrenergic receptors play an inhibitory role in hepatic steatosis in HFD-induced steatosis in mice.

## 4. Materials and Methods

### 4.1. Substances and Treatments

HFD 32 contained 25% proteins, 29% carbohydrates, and 32% fats (containing saturated, monounsaturated, and polyunsaturated fatty acids at 7, 22, and 4 g/100 g chow, respectively) [30]. The HFD had a calorific value of 507 kcal/100 g. The fat-origin calorific rate was 60% of gross energy. Phenylephrine hydrochloride, isoproterenol hydrochloride, and Triton WR-1339 were purchased from Sigma-Aldrich (St. Louis, MO, USA).

### 4.2. Animal Model and Experimental Design

Six-week-old male C57BL/6 mice were purchased from CLEA Inc. (Tokyo, Japan). After a 1-week acclimatization period of feeding on a basal diet (Oriental Yeast, Tokyo, Japan), the mice were divided into four groups (5–7 mice/group), and osmotic minipumps (Alzet model 2006; Alza, Palo Alto, CA, USA) containing phenylephrine, isoproterenol, or saline were implanted using an intercapsular incision into the subcutaneous space on the back under isoflurane anesthesia. Next, the mice were fed with a control diet or HFD, and phenylephrine (10 mg/kg/d), isoproterenol (30 mg/kg/d), or saline (3.6 μL/d) was coadministered continuously through the minipumps for five weeks. Doses of phenylephrine and isoproterenol were determined in accordance with those used in previous studies [31,32]. All mice were given free access to water and experimental diets. The body weights of the mice in each group were recorded weekly. Protocols regarding the use of mice were approved by the Institutional Animal Care and Use Committee of Aichi Medical University (Approved number: 2013-54, date of approval 26 November 2013). The handling of mice was in accordance with the National Institutes of Health “Guide for the Care and Use of Laboratory Animals”. After being fed with the experimental diets for five weeks, the mice were euthanized through CO_2_ inhalation. The livers were rapidly excised, fixed in buffered formalin (10%), frozen in liquid nitrogen, and stored at −80 °C. Blood samples were collected from the left ventricle and centrifuged, and the serum was stored at −80 °C.

Furthermore, another four cohorts of mice were fasted overnight, anesthetized with 70 mg/kg ketamine and 7 mg/kg xylazine intraperitoneally, and then injected with 0.2 mL of Triton WR-1339 (10% (*v/v*) solution) to inhibit the degradation of serum lipoprotein in saline. Blood samples were collected from the tail vein at 0 and 2 h. During the entire period, the animals had free access to water. TG assays were performed using a triglyceride detection kit (Wako, Osaka, Japan). Assuming that the total blood volume in the mouse represents 3.5% of its body weight [33], the rate of TG accumulation in mg/kg/h was determined after inhibitor (Triton WR-1339) injection.

### 4.3. Biochemical Characterization of Serum and Tissue

As described previously [32], serum ALT and FBG levels were determined using commercially available kits (Wako). Serum IRI levels were measured using a mouse insulin ELISA kit (Funakoshi, Tokyo, Japan). The stored liver samples (100 mg) were lysed and homogenized using a polytron homogenizer (NS-310E; MicroTech Nichion, Tokyo, Japan) for 1 min in a 2-mL solution containing 150 mM NaCl, 0.1% Triton X-100, and 10 nM Tris. Hepatic TG, FFA, and β-hydroxybutyrate levels were measured using a triglyceride detection kit (Wako), free fatty acid detection kit (Wako), and β-hydroxybutyrate assay kit (Cayman Chemical, Ann Arbor, MI, USA), respectively. Serum TG and FFA levels were also measured using a triglyceride detection kit (Wako) and a free fatty acid detection kit (Wako), respectively.

### 4.4. Histopathological Examination

Five-micrometer-thick sections of the liver tissue samples that were originally fixed in formalin and embedded in paraffin were examined for all analyses, as described previously [32,34]. Oil Red O staining was performed using a standard technique to examine hepatic fat deposition. The Oil Red O positive-area was quantified in five randomly selected fields per section. The percentage of Oil Red O-positive area was measured with a computerized image analysis system using Image-Pro Plus version 4.5 (Media Cybernetics, Silver Spring, MD, USA).

### 4.5. Real-Time Polymerase Chain Reaction (PCR)

As described previously [32], the frozen liver tissues were homogenized using TRIzol reagent (Life Technologies, Tokyo, Japan), and RNA extraction was performed using the RNeasy Mini Kit (Qiagen, Tokyo, Japan). The isolated RNA was resuspended in 40 μL of RNase-free water and quantified by spectrophotometry (optical density at 260 nm) and low-mass gel electrophoresis (Invitrogen, Tokyo, Japan). The total RNA extracted was reverse-transcribed to cDNA using the High Capacity cDNA Reverse Transcription Kit (Applied Biosystems, Foster City, CA, USA), according to the manufacturer’s instructions. Real-time quantitative PCR was carried out using ABI StepOne Sequence Detection System (Applied Biosystems), and TaqMan PCR was carried out using TaqMan Gene Expression Assays (ACOX1, Mm01246834_m1; adrenergic receptor α1 (ADRα1), Mm00442668_m1; adrenergic receptor β1 (ADRβ1), Mm00431701_s1; apolipoprotein B (apoB), Mm01545150_m1; Atg7, Mm00512209_m1; CD68, Mm00839636_g1; Col1A1, Mm00801666_g1; CPT1, Mm00463960_m1; FASN, Mm00662319_m1; MTTP, Mm00435015_m1; SREBF1, Mm00550338_m1; PPARα, Mm00440936_m1; SQSTM1, Mm00448491_m1; TNF-α, Mm00443258_m1; TGF-β, Mm00441724_m1; and TIMP1, Mm00441818_m1) and TaqMan Universal PCR Master Mix (Applied Biosystems) according to the manufacturer’s instructions. TaqMan PCR was performed following the protocol described in a previous study [35].

### 4.6. Western Blotting

The liver tissues were homogenized in sa odium dodecyl sulfate (SDS) sample buffer, separated on 10% SDS-polyacrylamide gels, and electrotransferred to nitrocellulose membranes. After blocking with 5% nonfat dry milk in TBST buffer (10 mM/L Tris-HCl (pH 8.0), 150 mM/L NaCl, and 1% Tween-20), the membranes were probed with an anti-LC3 antibody (dilution 1:1000; Cell Signaling Technology, Danvers, MA, USA), an anti-p62 antibody (dilution 1:1000; MBL, Nagoya, Japan), an anti-ADRα1 antibody (dilution 1:1000; Abcam, Cambridge, UK), and an anti-ADRβ1 antibody (dilution 1:1000; Abcam, Cambridge, UK), followed by incubation with horseradish peroxidase-conjugated anti-rabbit immunoglobulin G secondary antibodies (1:2000; DAKO Japan, Tokyo, Japan). Antibody binding was then visualized with an enhanced chemiluminescence reagent (GE Healthcare, Tokyo, Japan). Protein bands were imaged using the LAS1000 gel documentation system (Fuji Film, Tokyo, Japan) and densitometrically analyzed using Image Gauge software (Fuji Film).

### 4.7. Statistical Analysis

All results are expressed as mean ± SE. Statistical analyses were performed using analysis of variance (ANOVA) or Student’s *t*-test to compare data from different treatment groups. A *p*-value less than 0.05 was considered statistically significant. Bonferroni’s posthoc test was performed to analyze differences between multiple groups.

## Figures and Tables

**Table 1:** Clinical characteristics of mice fed experimental diets.

Group	*n*	Body Weight Gain (g)	Subepididymal Fat Weight (g)	FBG (mg/dL)	IRI (µg/L)
Control	5	2.23 ± 0.45	0.23 ± 0.03	178 ± 25	1.14 ± 0.34
HFD	6	7.69 ± 0.21 ^a^	0.31 ± 0.03 ^a^	215 ± 18	1.27 ± 0.36
HFD + phenylephrine	6	6.08 ± 0.93	0.37 ± 0.07	239 ± 6.5	1.19 ± 0.43
HFD + isoproterenol	7	8.4 ± 0.37	0.13 ± 0.03 ^b^	242 ± 11	0.45 ± 0.09 ^a^

HFD, high-fat diet; FBG, fasting blood glucose; IRI, serum immunoreactive insulin. Data were analyzed using ANOVA. Values represent means ± SE. ^a^ *p* < 0.05 for each group compared to the control group. ^b^ *p* < 0.05 for each group compared to the HFD group.

**Table 2:** Hepatic and serum TG and FFA contents in mice fed experimental diets.

Group	*n*	Serum TG (mg/dL)	Serum FFA (mEq/L)	Hepatic FFA (mEq/L)	Serum Cholesterol (mg/dL)
Control	5	30.1 ± 2.4	0.62 ± 0.05	0.23 ± 0.02	82.1 ± 4.8
HFD	6	40.2 ± 2.8	0.60 ± 0.03	1.13 ± 0.11 ^a^	156 ± 28
HFD + phenylephrine	6	53.4 ± 6.8 ^b^	0.98 ± 0.09 ^b^	0.60 ± 0.07 ^b^	140 ± 6.3
HFD + isoproterenol	7	37.8 ± 4.5	0.56 ± 0.03	0.90 ± 0.12	150 ± 3.8

HFD, high-fat diet; TG, triglyceride; FFA, free fatty acid. Data were analyzed using ANOVA. Values represent means ± SE. The overall *p*-values for steatosis, activity grade, and fibrosis were less than 0.05. ^a^ *p* < 0.05 for control compared to HFD. ^b^ *p* < 0.05 for HFD compared to HFD with phenylephrine.
